# Edward Jenner

**Published:** 1922-03

**Authors:** Hy. Wingrove

**Affiliations:** Kingston-on-Thames


### CORRESPONDENCE.

[Correspondence on all subjects is invited, but we cannot in any way be responsible for the opinions expressed by our correspondents, who must give their name and address as a guarantee of good faith, but not necessarily for publication. Correspondents are reminded that brevity of style and conciseness of statement greatly facilitate early insertion.]

### Edward Jenner.

*To the Editor of* THE HOSPITAL AND HEALTH REVIEW.

SIR,—I have read with great interest in your excellent periodical, THE HOSPITAL AND HEALTH REVIEW, of the discovery of vaccination by Edward Jenner, by C. J. S. Thompson, Curator of the Wellcome Historical Medical Museum. If he is not aware of it, perhaps he would like to know that Edward Jenner studied at *St. George's Hospital* under that great surgeon, John Hunter, who was on the staff of that Hospital. He might also like to know that there is another of the Jenner relics in existence, viz., the skin of the first calf from which Jenner took lymph; this is in St. George's Hospital Pathological Laboratory.

As late Secretary of St. George's Hospital (retired September, 1916), I should have liked to have seen Jenner's connection with the old Hospital mentioned.

I believe there is no Hospital in England with so long a list of eminent medical and surgical men as St. George's, nor one having so many whose names are honoured in Westminster Abbey.

I am, Sir,

Yours truly,

Kingston-on-Thames. HY. WINGROVE.

The National Baby Week Council, which has done such good work in the education of public opinion on the value of maternity and child welfare work, is now installed in its new offices at 117, Piccadilly (corner of Down Street), W. 1. The 1922 celebrations are to be held during the first week of July.

### HOSPITAL AND SICK-ROOM REQUISITES.

Where surgical india-rubber goods are required for hospital or sick room, the manufactures of Messrs. J. G. Ingram & Son, Ltd., appear to be of undoubted serviceability and quality. Set forth in their illustrated list are such articles as enemas (including the "Sterilendum," which is sterilisable in its entirety), syringes and sprays for various uses, hot water bottles, breast relievers, breast bottles, air cushions, bandages, gloves and gauntlets. Leaflets dealing with the advantages of the "Agrippa" Patent Band Teat, "Eclipse" hot water bottle and "Sterilendum" enema can be obtained on application to Messrs. J. G. Ingram & Son, Ltd., at the London India-Rubber Works, Hackney Wick, London, E.9.

### NEW DENTAL REGULATIONS.

We have received from the Registrar of the Dental Board of the United Kingdom a copy of the new Regulations under the Dentists Act, 1921, with reference to registration and other procedure provided for by that Act. Detailed information is given as to the persons now entitled to be registered as dentists, and of the conditions under which dental mechanics and others may sit for the prescribed examination (the syllabus of which is set forth), the passing of which will qualify them for registration. The formalities to be complied with by dental companies and their directors are also given. Copies of the Regulations may be obtained from the Registrar, Dental Board of the United Kingdom, 44 Hallam Street, London, W.1, price 2s. 6d., or 2s. 8d. post free.

